# Retraction: X. Ma *et al.* Hybrid Endovascular Repair in Aortic Arch Pathologies: A Retrospective Study. *Int. J. Mol. Sci.* 2010, *11*, 4687–4696

**DOI:** 10.3390/ijms161226233

**Published:** 2015-12-18

**Authors:** Mark L. Richter, International Journal of Molecular Sciences Editorial Office

**Affiliations:** 1 Department of Molecular Biosciences, the University of Kansas, Lawrence, KS 66045, USA; richter@ku.edu; 2 MDPI AG, Klybeckstrasse 64, 4057 Basel, Switzerland; ijms@mdpi.com

We have been made aware that the figures and experimental data reported in the title paper [1] are duplicated in another publication by P. Bergeron [2]. MDPI is a member of the Committee on Publication Ethics and takes the responsibility to enforce strict ethical policies and standards very seriously. To ensure the addition of only high quality scientific works to the field of scholarly publication, paper [1] is retracted and shall be marked accordingly. We apologize to our readership that this went undetected until now.
